# Unusual Case of Renal Tuberculosis in a Patient With Unsuspecting Chronic Back Pain

**DOI:** 10.7759/cureus.15177

**Published:** 2021-05-22

**Authors:** Morgan E Kensinger, Kathleen Adams, Jignesh Shah, Mudassar Zia, Joshua Floyd

**Affiliations:** 1 Internal Medicine, University of Missouri - Kansas City School of Medicine, Kansas City, USA; 2 Nephrology, University of Missouri - Kansas City School of Medicine, Kansas City, USA; 3 Radiology, Truman Medical Centers, Kansas City, USA

**Keywords:** tuberculosis, renal tuberculosis, psoas abscess, spinal tuberculosis, chronic back pain

## Abstract

Chronic back pain is a common complaint in the United States. In patients from endemic areas, spinal tuberculosis should be a part of the differential diagnosis, especially after the failure of conventional pain management treatments. Although most cases of tuberculosis present with pulmonary complaints, presented here is a case of isolated spinal tuberculosis with contiguous spread to the kidneys with the formation of psoas abscesses.

## Introduction

Despite its slowly declining incidence in the United States, tuberculosis remains a deadly and common infection worldwide. The CDC reports almost 9,000 provisionally reported tuberculosis cases in 2019 and an estimated 13 million living with latent disease in the United States [1]. Risk factors for tuberculosis include immigration from endemic areas and immunocompromising conditions, such as HIV and diabetes mellitus, amongst others. The diagnosis of tuberculosis is often delayed in the United States and other non-endemic regions, as course can be dependent upon the route and reactivity of infection, as well as the nonspecificity of symptoms evidenced by this case [2].

Other diagnostic challenges arise in uncommon or vague presentations, especially extrapulmonary disease, as discussed in this case. Spinal tuberculosis, or Pott’s disease, is an easily misdiagnosed condition. A 10-year (2002-2011) epidemiologic study of spinal tuberculosis in the United States found that, out of almost 76,000 patients diagnosed with tuberculosis, 3.7% had spinal tuberculosis [3]. Spread of spinal tuberculosis to contiguous tissues can result in psoas abscess formation. Despite frequent extensive disease, patients may often have very mild complaints, such as dull back pain or other nonspecific symptoms, and lack of signs of systemic infection [4]. Thus, care must be given by medical providers to not overlook fine details in patients with the aforementioned risk factors presenting with chronic back pain. This case discusses a Somalian male who presented with a two to three-year history of low back pain with subsequent diagnosis of spinal tuberculosis with formation of psoas abscess and contiguous spread to the left kidney.

## Case presentation

**Figure 1:**
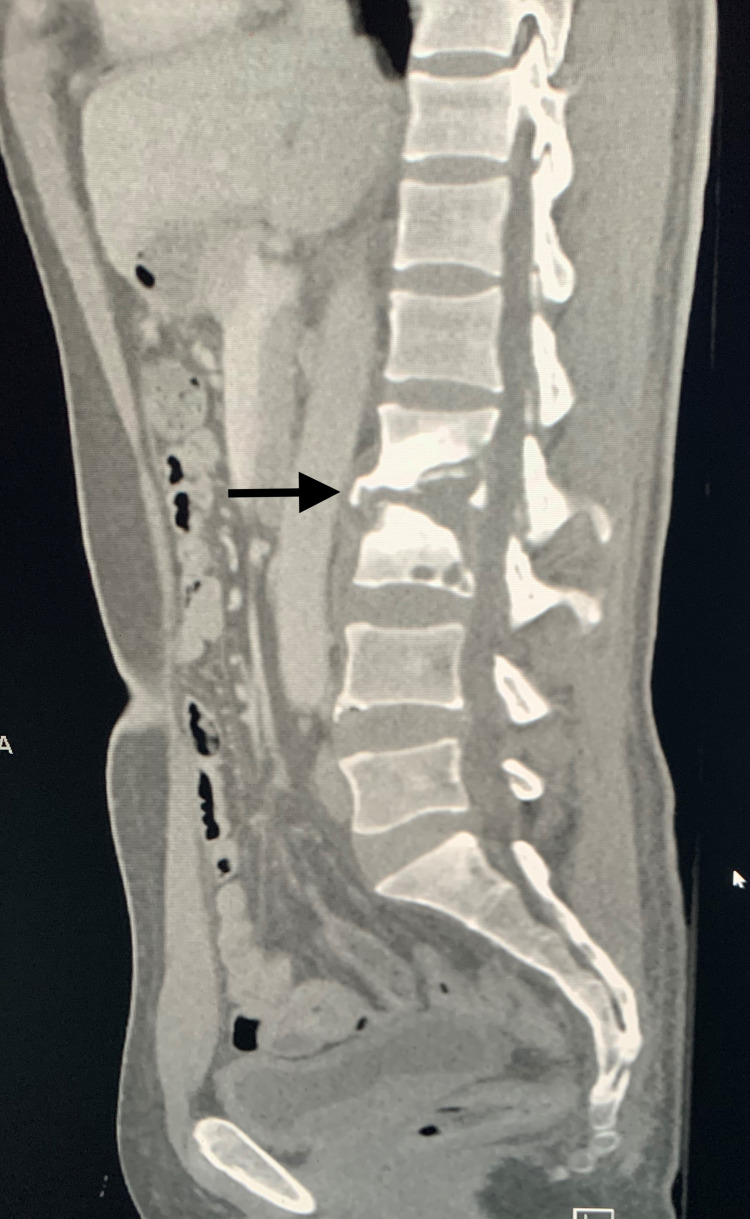
Sagittal CT of the abdomen at the level of the lumbar spine Sagittal image of the abdomen at the level of the mid lumbar spine utilizing bone windows. This demonstrates findings of chronic discitis/osteomyelitis at the L2/L3 level with evidence of a small prevertebral fluid collection.

A 46-year-old Somalian male who immigrated to the United States in 2016 presented to pain management clinic for complaints of chronic back pain ongoing for 2-3 years. His only known past medical history was type 2 diabetes mellitus. Family and surgical history were noncontributory. The patient had no known exposure to tuberculosis in the past. In the pain management clinic, the patient underwent multiple rounds of the sacroiliac joint and trigger point steroid injections for supposed degenerative changes in 2018 and November 2019 based on earlier imaging with only mild relief of back pain. Due to concerns for ongoing pain, a repeat MRI of the lumbar spine was ordered, which demonstrated discitis and osteomyelitis at L2-L3 with extradural collection in the spinal canal measuring 0.5 x 0.4 x 2.3 cm, as well as a collection anterior to the vertebral bodies at levels L2-L4 measuring 1.0 x 2.6 x 5.1 cm. Large bilateral psoas abscesses were also noted. CT was subsequently obtained inpatient. Figure 1 demonstrates the patient's discitis with an extradural collection. The patient was immediately referred to the emergency department for further evaluation.

The patient denied any history of fevers, chills, night sweats, and other symptoms of infection. Specifically, the patient denied any respiratory symptoms or complaints consistent with pulmonary tuberculosis. At initial evaluation, the patient denied back pain but complained of a two-day history of dysuria. White blood cell count was within normal limits at 4.30 x 10^3^ μL. Inflammatory markers were elevated with a C-reactive protein (CRP) of 6.5 mg/dL and erythrocyte sedimentation rate (ESR) of 64 mm/hr. He was diagnosed with an acute kidney injury (AKI) with a creatinine of 1.44; however, baseline creatinine was unknown. Urinalysis was positive for pyuria but with subsequent negative bacterial culture. There was a concern for spinal tuberculosis with psoas abscess formation due to MRI/CT findings and patient history of recent immigration. Quantiferon TB Gold test, urine culture and acid-fast bacillus (AFB), and blood culture and AFB were sent for microbiology evaluation.

**Figure 2:**
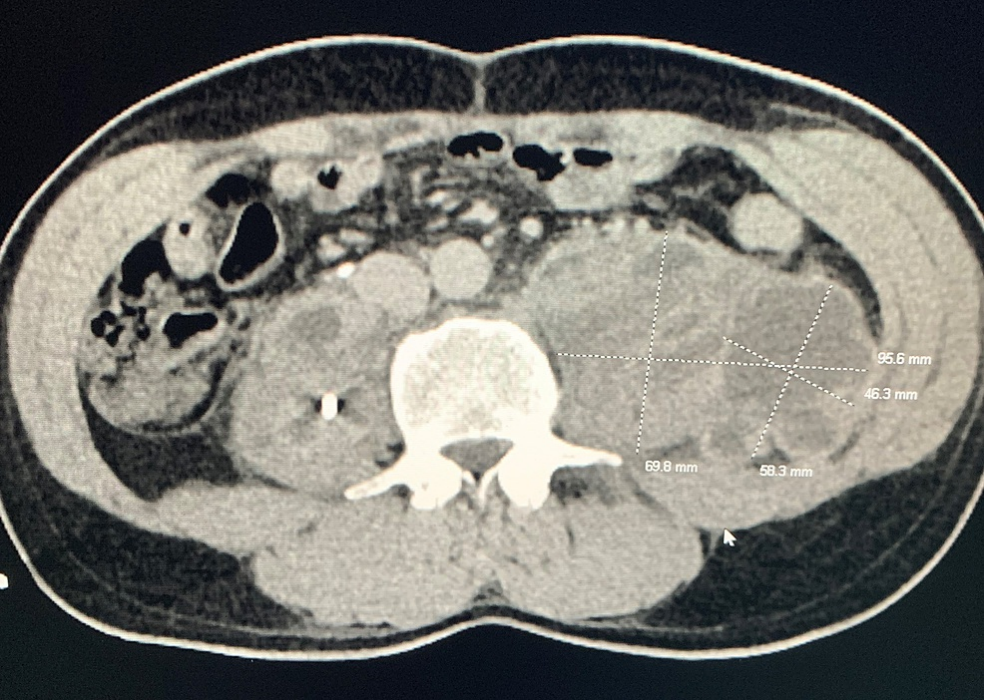
Axial CT demonstrating psoas abscesses with measurements Axial image of the abdomen at the level of the psoas muscles utilizing soft tissue windows. This demonstrates left greater than right psoas intramuscular complicated collections with thick intrinsic septations. There is a percutaneous drain present within the right psoas collection.

**Figure 3:**
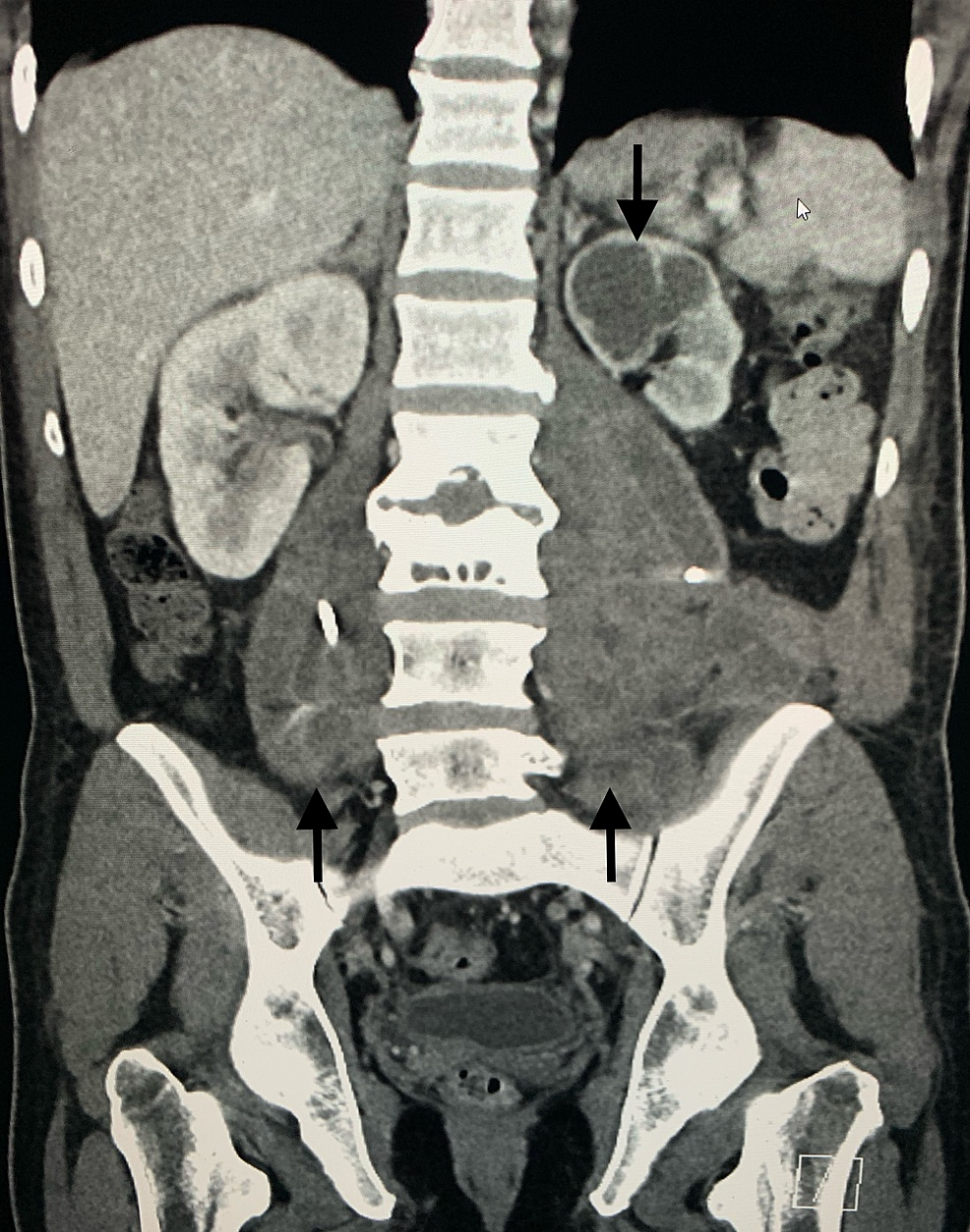
Coronal CT of the abdomen Coronal image of the abdomen at the level of the lumbar spine and kidneys utilizing soft tissue windows. This image demonstrates the extent of the bilateral psoas multiseptated collections. There is also asymmetric atrophy of the left renal superior cortex, a finding commonly seen with chronic renal TB. TB - tuberculosis

Quantiferon TB gold testing returned positive on hospital day 2. The patient underwent interventional radiology-guided psoas abscess drainage and bone biopsy. Chest X-ray was negative for pulmonary tuberculosis. Repeat abdominal CT scan showed inadequate drainage and drains were upsized. CT scan also demonstrated focal hydronephrosis of the superior left kidney with cortical thinning from the invasion of the left psoas abscess. Figures 2, 3 demonstrate the extent of psoas abscesses. Figure 3 additionally shows cortical thinning and left kidney cortical atrophy. Bone biopsy, urine, and blood cultures returned positive for *Mycobacterium tuberculosis* and empiric antibiotic therapy was discontinued with subsequent initiation of rifampin, isoniazid, pyrazinamide, and ethambutol therapy for the treatment of spinal tuberculosis. Adequate drainage of abscesses was obtained and bilateral drains were moved before patient discharge. The patient was discharged from the hospital after 15 days with plans to continue RIPE therapy for six months and strict follow up with Infectious Disease in the outpatient setting. The patient later had urine turn positive for AFB, suggesting psoas abscess led to renal tuberculosis due to proximity.

## Discussion

While uncommon in the United States, extrapulmonary tuberculosis must remain on the differential for unresolving chronic back pain, especially in patients with known risk factors. Increased awareness and consideration of spinal tuberculosis will have vast impacts on medical spending and patient outcomes, as it has been shown that delayed diagnosis is directly correlated to disease severity and disability [5]. As evidenced by this case, patients with spinal tuberculosis may be subjected to unnecessary procedures, injections, and/or physical therapy as methods of pain management with little to no benefit. Methods must be established for earlier diagnosis. Non-invasive tests that could lead to narrowed back pain differentials and expedited diagnosis of spinal tuberculosis include sputum and HIV testing, ESR, CRP, platelet count, white blood cell count, and alkaline phosphatase [6]. The Gold standard for diagnosis is CT-guided biopsy, however, this may not always be feasible in rural settings [7]. Directed history-taking and physical exam are the most significant and impactful tools in early diagnosis.

Diagnosis-limiting factors include lack of pulmonary and/or systemic symptoms and the low incidence of spinal tuberculosis. Overcoming these limitations is largely up to the medical provider in examining all patient factors. Although this diagnosis is not limited to immunocompromised patients, this population comprises a large portion of tuberculosis cases and subsequently spinal tuberculosis cases. Further consideration must be given to immigrated patients presenting with long-standing, unresolving back pain.

It is rare to see both spinal and renal tuberculosis in a single patient. Having psoas abscess bilaterally allowed for contiguous spread due to proximity. Fortunately, earlier diagnosis led to the timely management and resolution of these pathologic conditions.

## Conclusions

Spinal tuberculosis is often complicated by contiguous spread and formation of psoas abscesses. Diagnosis is often delayed due to low incidence, absence of pulmonary symptoms/findings, and mild back pain. The responsibility lies on medical providers to consider patient risk factors, exposures, and origins to accelerate diagnosis and lessen disease severity.

This case documents an uncommon cause of back pain and demonstrates multiple contributory factors to delayed diagnosis. While diagnosis remains a challenge, raising provider awareness, maintaining a high index of suspicion, and understanding patient risk factors may help overcome diagnostic challenges and lead to better patient outcomes.
